# Double-Step Machine Learning Based Procedure for HFOs Detection and Classification

**DOI:** 10.3390/brainsci10040220

**Published:** 2020-04-08

**Authors:** Nicolina Sciaraffa, Manousos A. Klados, Gianluca Borghini, Gianluca Di Flumeri, Fabio Babiloni, Pietro Aricò

**Affiliations:** 1 Department of Molecular Medicine, Sapienza University of Rome, Piazzale Aldo Moro, 5, 00185 Rome, Italy; gianluca.borghini@uniroma1.it (G.B.); gianluca.diflumeri@uniroma1.it (G.D.F.); fabio.babiloni@uniroma1.it (F.B.); pietro.arico@uniroma1.it (P.A.); 2 BrainSigns srl, Lungotevere Michelangelo, 9, 00192 Rome, Italy; 3 Department of Psychology, The University of Sheffield, International Faculty, City College, 54626 Thessaloniki, Greece; mklados@gmail.com; 4 IRCCS Fondazione Santa Lucia, Neuroelectrical Imaging and BCI Lab, Via Ardeatina, 306, 00179 Rome, Italy; 5 College of Computer Science and Technology, Hangzhou Dianzi University, Xiasha Higher Education Zone, Hangzhou 310018, China

**Keywords:** high-frequency oscillations, HFO, machine learning, epilepsy, intracranial EEG

## Abstract

The need for automatic detection and classification of high-frequency oscillations (HFOs) as biomarkers of the epileptogenic tissue is strongly felt in the clinical field. In this context, the employment of artificial intelligence methods could be the missing piece to achieve this goal. This work proposed a double-step procedure based on machine learning algorithms and tested it on an intracranial electroencephalogram (iEEG) dataset available online. The first step aimed to define the optimal length for signal segmentation, allowing for an optimal discrimination of segments with HFO relative to those without. In this case, binary classifiers have been tested on a set of energy features. The second step aimed to classify these segments into ripples, fast ripples and fast ripples occurring during ripples. Results suggest that LDA applied to 10 ms segmentation could provide the highest sensitivity (0.874) and 0.776 specificity for the discrimination of HFOs from no-HFO segments. Regarding the three-class classification, non-linear methods provided the highest values (around 90%) in terms of specificity and sensitivity, significantly different to the other three employed algorithms. Therefore, this machine-learning-based procedure could help clinicians to automatically reduce the quantity of irrelevant data.

## 1. Introduction

High-frequency oscillations (HFOs) have been proposed as biomarkers of the epileptogenic zone in the brain [1]. So far, HFOs have been differentiated in two different groups: ripples, oscillations in the range between 80 and 250 Hz, and fast ripples, oscillations in the range between 250 Hz and 500 Hz. The main asset of HFOs is not only their clinical application for presurgical identification of the seizure onset zone (SOZ, which can be considered as a close topological estimate of the epileptogenic zone [2]) but also as a predictor of clinical outcomes of cortex resection [3].

Even if visual inspection of intracranial electroencephalography (iEEG) signals for the detection of HFOs is still the gold standard, this method is not without pitfalls. In fact, it has been shown that it is highly time-consuming (10 min of 10-channel recording takes about 10 h [4]), subject-dependent and prone to errors due to the human factor [5]. For these reasons, an automatic and objective HFO detection algorithm is urgently required. 

The first examples of automated HFO detectors fall within *energy-thresholding-based methods*, considering that each HFO stands out from iEEG signals [6]. First, Staba et al. [7] defined the threshold based on the root mean square (RMS) of the bandpass-filtered signal. Second, Gardner et al. [8] defined the threshold according to the empirical cumulative distribution of short-time line length. Third, Crepon et al. [9] identified the threshold for HFO detection in five standard deviations of the envelope computed by Hilbert transform. The first distinction that can be made among these detectors is between parametric [7,9] and non-parametric threshold definition. The latter is the most preferable because no assumptions are made regarding the normal distribution of the signal. 

Hypothesizing that HFOs are rare events, threshold computation using the whole signal without distinguishing between events (HFO) and baseline segments (no-HFO), could be problematic for those channels where background activity is not perfectly flat, as for those channels where a large number of interictal epileptiform discharges (spikes) are present [6], since this could induce a high rate of false positive estimation, decreasing specificity. Therefore, it is essential to take into account the background activity to enhance specificity. The first algorithm in this direction was the Montreal Neurological Institute (MNI) detector [6]. Compared to standard thresholding methodologies, it computes the threshold by using only baseline segments. Consequently, a second issue arises, regarding how to automatically define the baseline. To overcome this problem, different solutions have been proposed. For example, the MNI detector identifies the baseline using wavelet entropy (WE), which measures the randomness of EEG signals [10]: each segment that is above the computed threshold [6] is considered as background activity. The baseline detection could be negatively affected when many HFOs are present. Therefore, if the initial hypothesis, that HFOs are rare events, is not valid, the baseline detection becomes less accurate and different methods need to be used [11].

Alongside the detection of HFOs, another issue is their classification to pathological or normal or even more to artifact. In fact, if the automatic detection of HFOs is intrinsically affected by low signal-to-noise ratio, non-stationarity, and by spikes or artifacts, HFO classification suffers from signals’ processing steps, in particular from pitfalls of classical filtering techniques (e.g., Gibbs phenomena). Indeed, it has been already evidenced that HFOs, which cover a wide range of frequency bands from the gamma (>40 Hz) up to the fast ripple (500 Hz) band, are induced under physiological conditions like some learning- or memory-related tasks [12]^,^ and sleep phases. These physiological HFOs are characterized by higher mean frequency, lower mean amplitude, and higher mean duration [13] compared to pathological ones. Therefore, the identification of pathological HFOs, indispensable for SOZ localization, is an example of a highly multiclass problem. It deals with distinguishing physiological HFOs’ high-frequency artifacts, false oscillations due to filtering of sharp transients, from pathological HFOs, which in turn form a group consisting of different possible subgroups (e.g., ripples, fast ripples, and fast ripples during ripples). Consequently, the ideal detector must face a very high number of variables to discriminate between all these possible classes. In this context, the employment of artificial intelligence methods could be the missing piece to achieve the goal of reliable detection and classification of HFOs.

Artificial intelligence (AI) could be considered as a tool [14], and one of the main applications of this tool is to support humans in decision-making. This is possible thanks to the ability of agents (i.e., every kind of machine or instrument) to learn by means of input data and, therefore, to make a decision to achieve a goal, since for humans it is quite impossible to manage a high volume of data and variables, whereas computer-based instrumentation does it effortlessly. Machine learning is a subset of AI, including the set of algorithms that allow machines to learn [15]. Though the ways of learning can be different, any approach is based on preliminary identification of the most relevant aspects of data used by the machine to learn: the features. To avoid spending too much time in designing and selecting features, the choice of features should be made a priori to provide the model only relevant and coherent information necessary for learning. In contrast, deep learning algorithms, which have become very popular in recent years, could be feed by a high number of features, without physical meaning and obtained bypassing the human engineering step [16]. These are usually employed in image recognition, where they demonstrated to be a powerful but blind method. Indeed, after network training it is very difficult to get back into the feature-set used to define the model. For these reasons, this study was based on machine learning algorithms, which are more transparent and explainable compared to deep learning ones [17].

One of the first examples of machine learning algorithms applied to HFO detection has been provided by Blanco et al. [18]. They employed an unsupervised cluster analysis to differentiate HFOs according to their characteristics, in order to avoid the low inter-rater reliability of HFO visual marking. In particular, for each HFO, they computed seven different features that allowed them to find four different clusters: three different clusters of HFOs (ripples, fast ripples, and ripples + fast ripples) and one for artifacts. Therefore, they confirmed that HFO classification is a multiclass problem. Subsequently, different classification methods have been tested: LDA [19], support vector machine (SVM) [13,20,21], and decision tree [22], combined with different feature sets.

To increase the separability between HFOs and interictal epileptic spikes, Jrad et al. [19] used the energy ratio between bands computed with discrete wavelet transform as features. A multiclass LDA has therefore been trained and provided median sensitivity greater than 0.85 and a median specificity >0.95 for all four classes. 

Considering that several different kernels can be used, SVM represents a very suitable method for HFO detection, and this is why it is the most used method in this context. A radial basis function SVM [20] has been trained with RMS and the energy ratios of HFOs obtained by means of a Gabor transform, providing a sensitivity of 0.92 for ripples and 0.73 for fast ripples and a specificity of 0.738 for ripples and 0.933 for fast ripples. Moreover, linear SVM with cross-subject validation has been used to binary classify pathological from physiological HFO, using spectral amplitude, frequency and duration features and providing a sensitivity ranging from 68% to 99% and specificities of >90% [13]. Again, a linear SVM has been used to discriminate real HFOs from false HFOs caused by filtering of sharp events, providing sensitivity and specificities greater than 70%, applying a leave-one-subject-out validation [21]. In this case, raw, unfiltered data has been used to compute 26 temporal features selected by forward feature selection (actually, only three features have been used) on a different subset of subjects. 

A decision tree classifier has been trained with six features related to energy and duration obtained from a time frequency analysis and provided a mean sensitivity of 66.96% [22]. To compare this relative lower value of sensitivity, the same work compared on the same dataset five other different approaches (RMS [7], CMOR [23], bumps [24], matching pursuit [25] and HHT [26]). They found that the HHT-based method represents the best compromise between sensitivity and false discovery rate (FDR); however, decision tree provided the lowest value of FDR [22]. The authors claimed that low FDR is desirable in the case of the fully automatic procedure, because it is safer with respect to the high number of false HFOs that could be related to filtering of the sharp transients. However, other evidence suggests that, for clinical application, it may not be necessary to separate real HFOs from false oscillations produced by the filter effect of sharp spikes [27].

Alongside the employment of traditional temporal and spectral features used for HFO description, Firpi et al. [12] proposed to use artificial/data-driven features that might not have a physical meaning, however, are more sensitive to HFO variability. PSO-NN (Particle Swarm Optimization- Neural Network) is an algorithm employed to detect patterns that allow the distinction of HFOs from background, using a k-nearest neighbors (KNN) classifier. Moreover, recently HFOs have been directly used as features with other biomarkers of the epileptogenic zone to classify the electrodes defining the SOZ [28].

Even if our work has been focused on machine learning methodologies, deep learning attempts at HFO detection are noteworthy. A radial basis neural network has been used to detect ripples in a cross-subject evaluation [4]. It obtained a sensitivity of 49.1% and a specificity of 36.3%. In this case, three features were used: line length, energy, and instantaneous frequency. An artificial neural network has been trained using approximate entropy to classify HFOs from background activity in four rats [29]. Approximate entropy has been used as a feature to identify HFOs with respect to baseline segments: during an HFO, the complexity of the signal is higher, therefore its approximate entropy is higher. Finally, a convolutional neural network (CNN) has been used for automatic detection of ripples and fast ripples [30]. The CNN’s performance has been compared with the four reference-detectors collected in the RIPPLELAB application [31] and showed much higher sensitivity (77.04% for ripples and 83.23% for fast ripples) and specificity (72.27% for ripples 79.36% for fast ripples) except than the specificity of Staba detector [7] for ripples (83.14%) and the sensitivity of the MNI detector [6] for fast ripples (74.77%). In Table 1 some of the works described so far are summarized.

In this work, we propose a double-step procedure based on machine learning algorithms to both detect and classify HFOs. In particular, only features related to energy were employed, because signals have been pre-segmented with windows of different duration according to the standard duration of the events of interest. Furthermore, we aimed to classify three different kinds of HFOs (ripples, fast ripples, fast ripples co-occurring during ripples). In fact, according to [27], fast ripples occurring during ripples are usually erroneously considered ripples, even if it has been demonstrated this is an important class, because fast ripples during ripples provide more accurate SOZ estimation than fast ripples and ripples separately [32]. 

## 2. Materials and Methods

### 2.1. Dataset Description

The dataset was downloaded from the CRCNS.org platform [33]. It contains iEEGs acquired at 4000 Hz sampling frequency and then down-sampled to 2000 Hz from 18 patients during sleep. The dataset provides, for each subject, a differential montage, and each HFO was marked using the automated algorithm described in [34] in three different classes: ripples, fast ripples and fast ripples co-occurring during ripples [32]. The importance of the latter class is related to the already-demonstrated high correlation with seizure outcome. Therefore, the dataset provides start and end times of each event, and this information was used as ground truth to label each segment. As encouraged by a recent review [35], we performed the analysis on an individual patient basis. 

### 2.2. Feature Extraction

For each subject, 5 min of interictal recording was used. Each session was filtered in three different bands (ripples band 80–250 Hz; fast ripples band 250–500 Hz; overall HFOs band 80–500 Hz) and then divided into segments. In particular, windows of 10, 50 and 100 ms were used according to the mean durations of ripples (96.2 ± 45.5 ms) and fast ripples (40.6 ± 26.7 ms) found in [36] and HFOs in general (30–100 ms) [37]. For each band and each segment, four features were computed:

- Line length according to the [8] definition;
- Short time energy according to the [4] definition;
- Root mean square (RMS) according to the [7] definition;
- Teager energy according to [22].

Such energy features allow having in one model the traditional energy-thresholding-based method characteristics [4,7,8] and the nonlinear information provided by Teager energy, whose advantage is to detect instantaneous frequency and amplitude [22]. We used only energy features, because they do not need information regarding the whole duration of the signal [29] and have low computational cost [38] in view of a future real-time application. Moreover, the energy computation in 5 and 10 ms windows has been already validated by [39,40].

### 2.3. Machine Learning Algorithms

The proposed procedure is based on two steps:

1. HFO detection
2. HFO classification.

In both phases, five different machine learning algorithms were employed to cover a wide range of algorithm categories. Each of them was used twice: in binary configuration to discriminate HFO segments and no-HFO segments (first step) and in multiclass configuration to classify ripples, fast ripples and fast ripples co-occurring during ripples among HFOs (Second step). In particular, the following algorithms were trained on the aforementioned energy features:

- Linear discriminant analysis (LDA) [41,42] is a linear algorithm that allows creating hyperplanes in n-dimensional space according to number of features, to discriminate two or more classes. In this case it has been used without hyperparameters to optimize;
- Logistic regression (LR) [43] is a regression-based method employed to predict the probability of occurrence of an event. In this case, the value of l2 penalization has been chosen in log space between −3 and 3;
- Support vector machine (SVM) [44] is a supervised algorithm that allows creating hyperplanes in n-dimensional space according to the number of features, to discriminate two or more classes. In this case it has been used a linear kernel and the optimal cost parameter has been chosen in a log space between −3 and 3;
- K-nearest neighbors (KNN) [45] is a nonlinear instance-based algorithm. Its main idea is to predict the class based on distance between the observation and the first k neighbors and does not assume a priori the dataset distribution. The number k of neighbors has been chosen in a range from 1 to 20;
- Random forest classifier (RF) is a nonlinear classifier [46] belonging to the ensemble methods. This family of classifiers makes it possible to generalize well to new data [47] and they are more robust to overfitting than individual trees because each node does not see all the features at the same time [46]. In this case, the number of trees (100, 200), the maximum number of levels in tree (5, 10, 20), the minimum number of samples required to split a node (2, 5, 10), and the minimum number of samples required at each leaf node (1, 2, 4) have been chosen for optimization.

#### 2.3.1. Step 1: HFO Detection

For HFO detection with respect to the segments without HFOs, binary classification was employed. Five different classifiers (LDA, logistic regression, SVM, KNN, random forest) were calibrated on the set of twelve features already described. Due to the high class imbalance (the number of segments with HFOs was less than 10% of the total number of observations), the number of observations was set according to the minimum number of segments labelled like HFOs that were variable over the subjects (on average 30000), but also, in the worst case (3600), due to the low number of features, we can assure that the training set is at least 10 times higher than the number of features itself in order to avoid overfitting. The segments were randomly selected. A total of 30% of observations were used as a validation set for hyperparameter optimization for four of the five classifiers (LDA has been considered without optimization). The remaining 70% were used in a 5-fold cross-validation to analyze how much each classifier was able to generalize during HFO detection for each patient. The average performances obtained for each subject are described in terms of area under curve (AUC), sensitivity, and specificity. They were statistically compared by performing a Friedman test (α = 0.05) and, if necessary, the Nemenyi post-hoc test, using the R package described in [48].

To further assess the practical employment of automatic detection, it is necessary to analyze its capacity to generalize and its complexity. To reach this aim, for each subject, a 10-fold cross-validation was performed on a limited number of observations [49,50]. Each model was trained on a variable number of observations: 720, 1440, 2520, and 3240. For each level was computed (i) the AUC obtained from training and validation and (ii) the time necessary to train the model. 

This step made it possible to choose the best configuration of algorithm and window length for HFO detection.

#### 2.3.2. Step 2: HFO Classification

After identification of the best window length for HFO detection, multiclass classifiers were calibrated, with the feature set already described, to be able to classify among ripples, fast ripples, and fast ripples co-occurring with ripples. Additionally, in this case, 30% of observations were used as a validation set for hyperparameter optimization and 70% in 3-fold cross-validation. The ripples class had the highest number of observations (90% of the total); therefore, to balance the observations of the other two classes, ADASYN oversampling procedure [51] was applied. The performance of the classification has been described in terms of sensitivity and specificity. To statistically compare these values, a Friedman test was performed, and according to [52] Bergmann and Hommel’s procedure was adopted for multiple pairwise corrections of p-values. This approach provides more powerful statistical results and is recommended for comparison of less than 10 algorithms. 

## 3. Results

### 3.1. Step One Results

**Figure 1:**
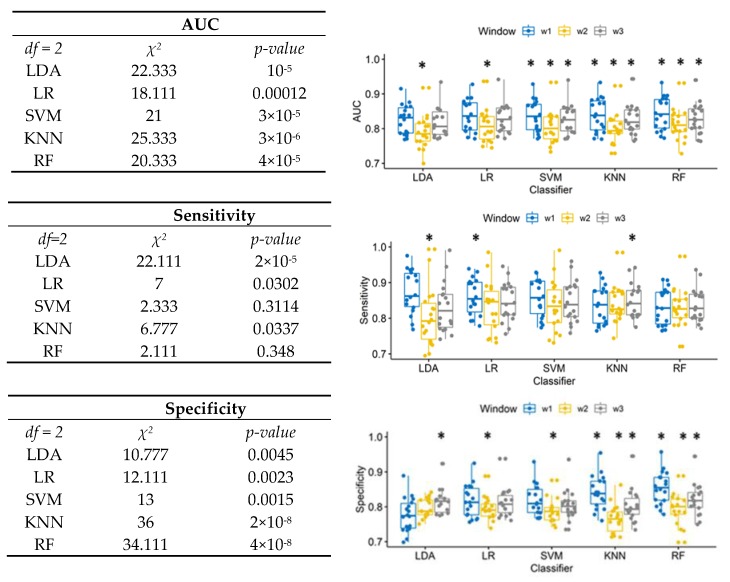
Left: Friedman test results to compare window effects for each classifier in terms of AUC, sensitivity, and specificity. Right: box plot of the AUC, sensitivity, and specificity for different segmentations (win1 = 10 ms, win2 = 50 ms, win3 =100 ms). The asterisks * showed the post-hoc results.

Figure 1 shows on the left the results of Friedman test performed for each classifier separately, to compare the performance when different window lengths were used, and in the boxplot on the right the results of related Nemenyi post-hoc test. For all classifiers, 50 ms window length provided significantly lower AUC. For SVM, KNN, and RF, 10 ms window provided the highest AUC. 

The sensitivity of SVM and RF was not affected by window length. The 50 ms window length provided the lowest sensitivity for LDA. The significantly highest sensitivity for LDA and KNN was provided, respectively, by the 10 ms window and 100 ms windows. 

The window effect on specificity was significant for all classifiers. The 50 ms window provided the significantly lowest specificity for LR and SVM. For LDA, the highest specificity was provided by the longest window. For KNN and RF, the shortest window also provided the highest specificity. 

The 10 ms window provided the highest value of AUC relative to the wider windows, between 0.825 and 0.843: according to results of the Friedman test (Friedman’s chi-squared = 53.911, *df* = 4, *p*-value < 10^−11^), LDA provided the lowest AUC (0.825), and RF has the significantly highest performance (0.843). Moreover, according to the classification method, the 10 ms window is also able to provide the highest sensitivity (0.874 for the LDA) and the highest specificity (0.854 for RF).

**Figure 2:**
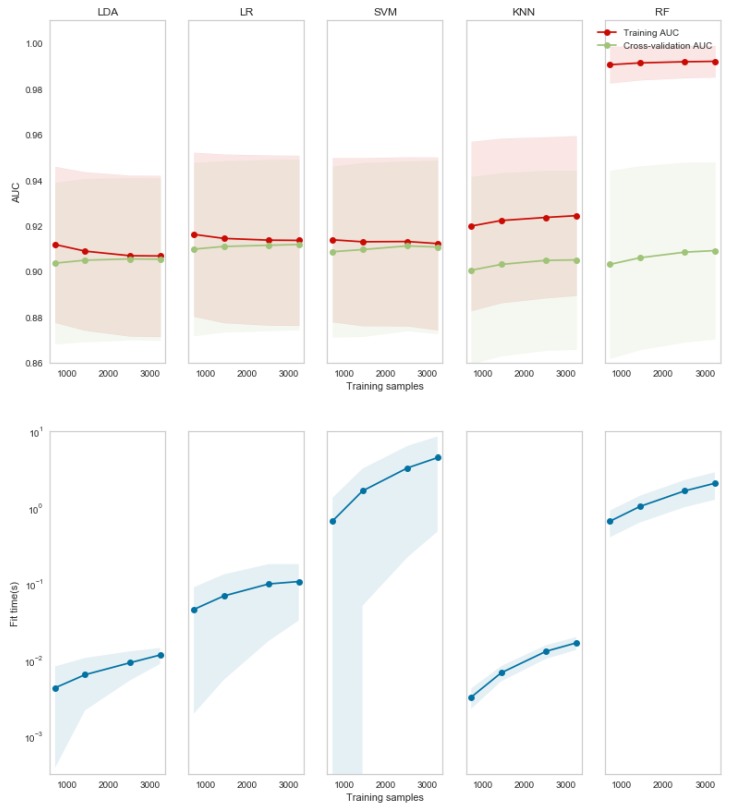
AUC (first row) and time for training (second row) for each of the five investigated algorithms (in each column) with different numbers of observations. The lines represent the population averages and the shadows the standard deviation.

To analyze the effective employment of the 10 ms window, it was used for training each method with a low number of observations. In Figure 2, in the first row the red lines show the value averaged over all subjects obtained during training and the green lines those obtained by 10-fold cross-validation. The shadows represent the standard deviation for the population. For LDA, LR and SVM there was a convergence of AUC values for less than 3000 samples, showing a good bias-variance trade-off. However, such a number of observations is not enough for both KNN and RF, which still showed high variance. Analyzing the time necessary to train the model, due to the absence of parameters to optimize, LDA was on average more than 10 times faster than LR and SVM.

In order to define a model that is reliable, but also practical to use, LDA could be chosen because it allows the fastest training (both in terms of fit time and number of observations). Moreover, according to the results in Figure 1 for LDA, the 10 ms window provided a not significantly lower AUC, but the highest sensitivity of HFO detection. The choice of the classifiers could be made according to these results, and the 10 ms segmentation was chosen for the second step, i.e., the classification of the three classes: ripples, fast ripples and fast ripples co-occurring with ripples.

### 3.2. Step Two Results

**Figure 3:**
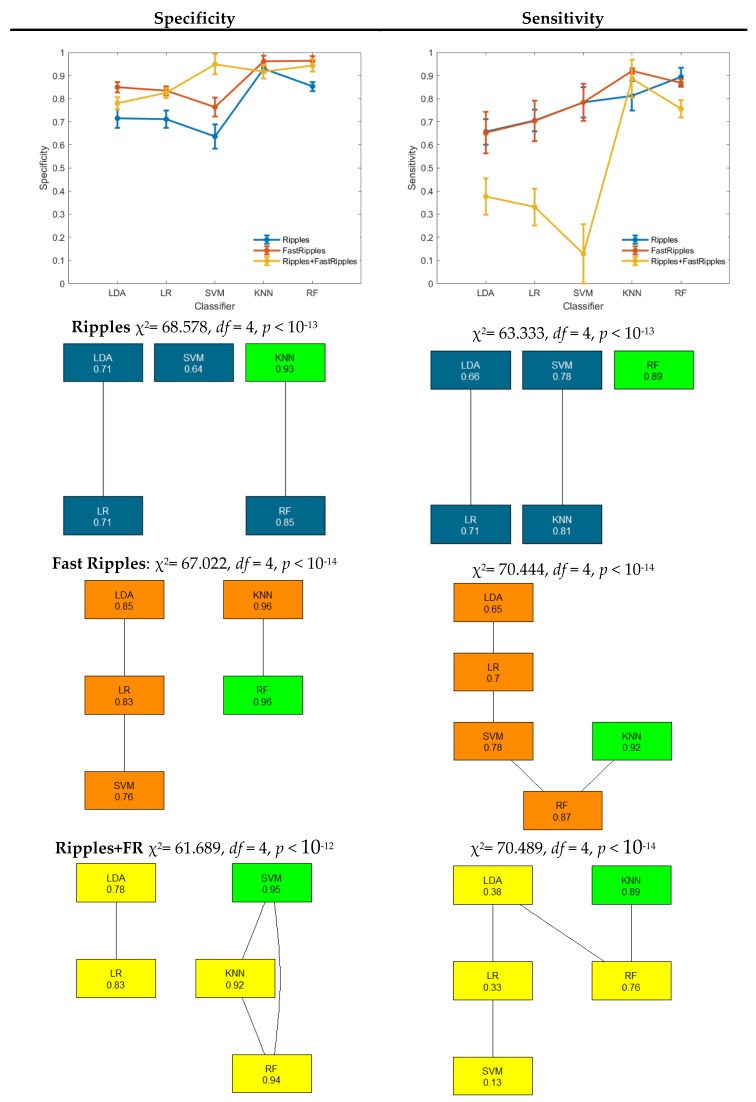
Three-class classification performance in terms of specificity and sensitivity for each class. The results of the Friedman test have been reported above the Bergmann and Hommel’s procedure graph.

In Figure 3, specificity and sensitivity of the three different HFO classes are shown. For each class, a Friedman test has been performed, and post-hoc results corrected with Bergmann and Hommel’s procedure are shown by means of a graph. Each node represents an algorithm, and the connection represents the fact that the difference between such algorithms is not significant. The algorithms with the highest performance have been highlighted in green. The results related to the ripples class are in blue, those related to fast ripples in orange and those related to fast ripples occurring during ripples are in yellow.

RF provided the significantly highest sensitivity in ripple detection (0.89). The highest specificity has been instead provided by KNN, which, however, is not significantly different from RF specificity which is 0.85. Additionally, for fast ripple detection, KNN and RF provided the highest specificity, significantly higher with respect to the other three algorithms. 

Concerning sensitivity, KNN provided the highest value (0.92), which is not significantly different from the RF sensitivity (0.87). The detection of the class fast ripples co-occurring with ripples provided the lowest value of sensitivity. In this case, SVM provided the highest specificity, which is not significantly different from KNN and RF specificity. Again, KNN provided the highest sensitivity that is not significantly different from that of RF (0.76).

## 4. Discussion

Visual detection of HFOs is a highly time-consuming activity, and the employment of automatic detection has been proposed as a solution since 2002. Despite the evolution in automated detectors proposed so far, different open issues still affect automated detection. These issues are strictly linked to the high number of patterns to classify (i.e., HFOs vs. no HFOs, and different types of HFO), and consequently the complex multiclass problem. Most of the employed detectors suffer from high specificity and are not able to distinguish physiological HFOs, high-frequency artifacts, false oscillations due to the filtering of sharp transient from pathological HFOs that is a category consisting itself in different possible groups (e.g., ripples, fast ripples, and fast ripples during ripples). In general, thanks to the employment of machine learning, it is not necessary to assume that HFOs are rare events and to define manually a threshold, therefore the typical high number of false positive of traditional automatic detectors decreases [53]. Moreover, recently, it has been highlighted that it is necessary to further refine HFO identification, differentiating HFOs with respect to the baseline from HFOs occurring during spikes, and defining different thresholds for SOZ identification, varying according to brain regions [54]. Finally, a recent review [35] suggested that researchers should go forward with a statistics-based patient-oriented research.

In this work, a double-step procedure has been proposed in order to detect HFOs and to classify the three classes already defined, comparing different Machine learning algorithms. Firstly, the iEEG signals have been divided into segments of different duration (10 ms, 50 ms, 100 ms) according to the mean duration of ripples and fast ripples found in the literature. According to the AUC values (Figure 1), a 10 ms window provided the best performance for distinguishing between HFO and no-HFO segments, always on average higher than 0.80 of AUC. Higher temporal resolution (i.e., employing short temporal windows for the analysis) allows to better separate between HFO and no-HFO probably because the longer is the time of observations, the more likely is to observe a contingency of events that could create a new cluster of energy values not associable with either HFOs or no HFOs. Among the algorithms, RF provided on average the best performance, which was 0.84. However, in this context, it is important to take into account the role of false positive and false negative rate. In clinical studies, higher sensitivity is preferable to higher specificity because it is necessary to control false negative responses with respect to false positives. For this reason, even if LDA provided the lowest AUC, it should be employed due to the higher value of sensitivity (0.874) compared to the other algorithms. Additionally, in this case, it has the shortest window allowing for the highest sensitivity. According to [49], reducing the window size translates into faster detection. Consequently, the shorter the window, the greater will be the number of observations for signals of the same duration. This aspect is of fundamental importance for practical application. In fact, in this work, we have been able to use all the available data because performance evaluation has been performed off-line to investigate the pros and cons of different methods. Even if model selection performed by means of statistical comparison is a procedure widely used in the literature [49,55,56,57], in a practical context, a limited number of samples for training could negatively affect performance and therefore model selection. Analyzing the performance of each algorithm as the number of samples vary, LDA showed a good bias-variance trade off, and its training and cross-validation performance converge for less than 3000 samples. Moreover, because it is not an optimized algorithm, it is 100 times faster to train relative to SVM. Therefore, it could be a good candidate for a practical application and with a view to testing a real-time application in the future.

It is quite difficult to compare these results with current literature because few works have reported applications of machine learning algorithms for HFO detection and, moreover, any work used high temporal resolution. However, one of the examples showed that, by using six features related to energy and duration, it was possible to discriminate HFOs from no HFOs with a sensitivity of 66.96% by using, in particular, a decision tree algorithm [22]. In another case, artificial features have been compared with the application of the classical RMS, showing significantly higher sensitivity [12].

Different multiclass machine learning techniques have at this point been compared, by using the 10 ms windowing previously identified, on HFO-related segments. This step aimed to distinguish among three classes, i.e., ripples, fast ripples, and fast ripples during ripples. The addition to classical ripple and fast ripple discrimination of the third class (i.e., fast ripples during ripples) has been taken into account for two reasons: the dataset we used as a benchmark allowed us to also define this class, and the results already obtained in this context showed that fast ripples co-occurring with ripples are highly correlated with the SOZ definition, more than ripples and fast ripples alone [34]. For all the classes (Figure 2), KNN and RF provided the highest values in terms of specificity and sensitivity (89% Sensitivity in RF for ripples, 92% in KNN for fast ripples and 89% in KNN for fast ripples occurring during ripples), which were, in general, significantly different with respect to the other three algorithms. No significant differences were found among them. In this case, it is possible to compare such results with few literature studies. For example, a multiclass LDA applied to distinguish between ripples, fast ripples, fast ripples co-occurring with ripples, and artefacts provided, on average, a sensitivity of 80.5%. This result was achieved by employing energy ratio features [19]. Another five-class problem showed a sensitivity of 73% for fast ripples and 92% for ripples employing an RBF-SVM [20]. In Table 2, the values of specificity and sensitivity of comparable works in the literature have been summarized and compared with the results obtained with this procedure. The obtained sensitivity is higher relative to the other methods, while the specificity is lower for HFO detection relative to no HFOs and higher for event classification. However, applying this whole procedure, it is possible to obtain both detection and classification of ripples, fast ripples and fast ripples co-occurring with ripples, other than the other works. 

Finally, the two-step procedure employed in this study showed that it is possible to detect HFOs with an accuracy higher than 82% and a sensitivity, on average, of 87%, using energy features computed on 10-ms wide segments and applying a linear classifier (i.e., the LDA). This is coherent with the nature of HFO identification and the features used because, according to what happens with traditional detectors, HFOs can be identified by an energy threshold criterion. In this case, only energy features were used because the duration was fixed by the extent of the window used for signal segmentation. Employing different features, introducing time and time-frequency information could help this detection, as it has been already demonstrated [26]. Notwithstanding the performance of the proposed approach, it provides higher sensitivity when compared to those obtained in the literature with other machine-learning-related approaches. Moreover, the second step showed that by employing energy features, it is possible to distinguish among ripples, fast ripples and fast ripples during ripples with sensitivity higher than 90%, a result possible thanks to the employment of KNN or RF methodologies. This could be explained by the fact that both are non-linear methods, and therefore present advantages concerning the other tested methods in case of multiclass problems. In particular, the KNN method is efficient for an online application when there are few features, and random forest tends to generalize well and is less subject to overfitting.

To the best of our knowledge, this is the first work that tries to detect HFOs with such high temporal resolution (in windows from 10 ms to 100 ms). This approach makes it possible to have high sensitivity almost all for short events like fast ripples (whose minimum duration has been found around 10 ms, and which are important because they provide higher specificity for SOZ definition [54]) and to face the broad morphological and spectral diversity of HFOs, both intra- and inter-subject [20]. Moreover, the high temporal resolution makes it possible to analyze the transient nature of the HFOs, which has been hypothesized to be a feature to allow the differentiation of physiological and pathological HFOs [58]. In fact, from a clinical point of view, in the state of art, the direct application of automatic detection of HFOs in clinical contexts is not yet possible due to the not-perfect accuracy of algorithms and the impossibility of detecting a pathological HFO with respect to physiological ones [35]. However, the proposed double-step procedure could be easily used by experts as an aid to dramatically reduce the quantity of irrelevant data, highlighting just the parts of signals that have been classified as ripples, fast ripples or fast ripples co-occurring with ripples. During the typical clinical procedure, clinicians should manually annotate offline hours of multichannel iEEG, which is a time-consuming procedure, which requires the full attention of the clinician, hence leading to missed HFOs and to wrong delineations of the SOZ. The presented approach has a major clinical impact, since it can be further developed into user-friendly software for the clinics that perform surgical operations for medical refractory epilepsy, in order to help clinicians to detect HFOs very quickly, without needing to spend lots of hours in manual annotation of EEG, leading to more accurate detection of SOZ, and increasing the probability of seizure freedom postoperatively. 

## 5. Conclusions

With this work, we have demonstrated that the application of machine-learning algorithms can efficiently be used to detect and classify HFOs. Assuming a high temporal sampling for this analysis, it has been shown that the computation of energy features allows a sensitivity of 87% for HFOs on average, by using linear discriminant analysis. Moreover, non-linear methods like KNN and random forest can provide a sensitivity of 89% for ripples, 92% for fast ripples and 89% for fast ripples occurring during ripples.

## Figures and Tables

**Table 1:** Applications of artificial intelligence for HFO classification.

AI Technique	Classes	Sensitivity	Features
KNN [12]	2 classes (HFO/ background)	NN features provide sensitivity significantly higher than RMS for 4/6 subjects.	RMS vs. data-driven feature extraction with NN
Multiclass LDA [19]	4 classes (ripple, fast ripples, ripple + fast ripples and artifacts)	Median 80.5%	Energy ratio computed with discrete wavelet
Decision tree [22]	2 classes (HFO/ no-HFO)	66.96%	6 features related to energy and duration
RBF SVM [20]	5 classes (gamma, high gamma, ripple, fast ripples and artifacts)	73% fast ripples 92% ripples	Energy ratio and root mean square features computed on Gabor transformed data.
Linear SVM [13]	2 classes (pathological/physiological)	Ranging from 68 to 99%	Spectral amplitude, frequency, and duration
SVM [21]	2 classes (false HFOs due to filtering effects during sharp events/real HFOs)	>70%	26 temporal features selected with forward feature selection.
Radial basis neural network [4]	Cross-subject ripple classification	49.1%	Line length, energy and instantaneous frequency
Convolutional neural network [30]	2 classes (ripples/no ripples and fast ripples/no fast ripples)	77.04% ripples 83.23% fast ripples	Grayscale images of iEEG amplitude

**Table 2:** Comparison of the obtained results with similar procedures in the literature.

	Sensitivity				Specificity			
	*HFO/no-HFO*	*R*	*FR*	*FR+R*	*HFO/no-HFO*	*R*	*FR*	*FR+R*
Our Work	0.874	0.89	0.87	0.76	0.776	0.85	0.96	0.95
[22]	0.669				0.913			
[30]		0.77	0.83			0.72	0.79	
[20]		0.91	0.72			0.73	0.93	
